# Supplementary material for: Sleep/Wake Behavior and EEG Signatures of the TgF344-AD Rat Model at the Prodromal Stage
Source: Int J Mol Sci. 2020 Dec 5;21(23):9290. doi: 10.3390/ijms21239290 (PMC7730237; doi:10.3390/ijms21239290)
Supplement: Supplementary file 1 [file ijms-21-09290-s001.pdf]

Appendix A

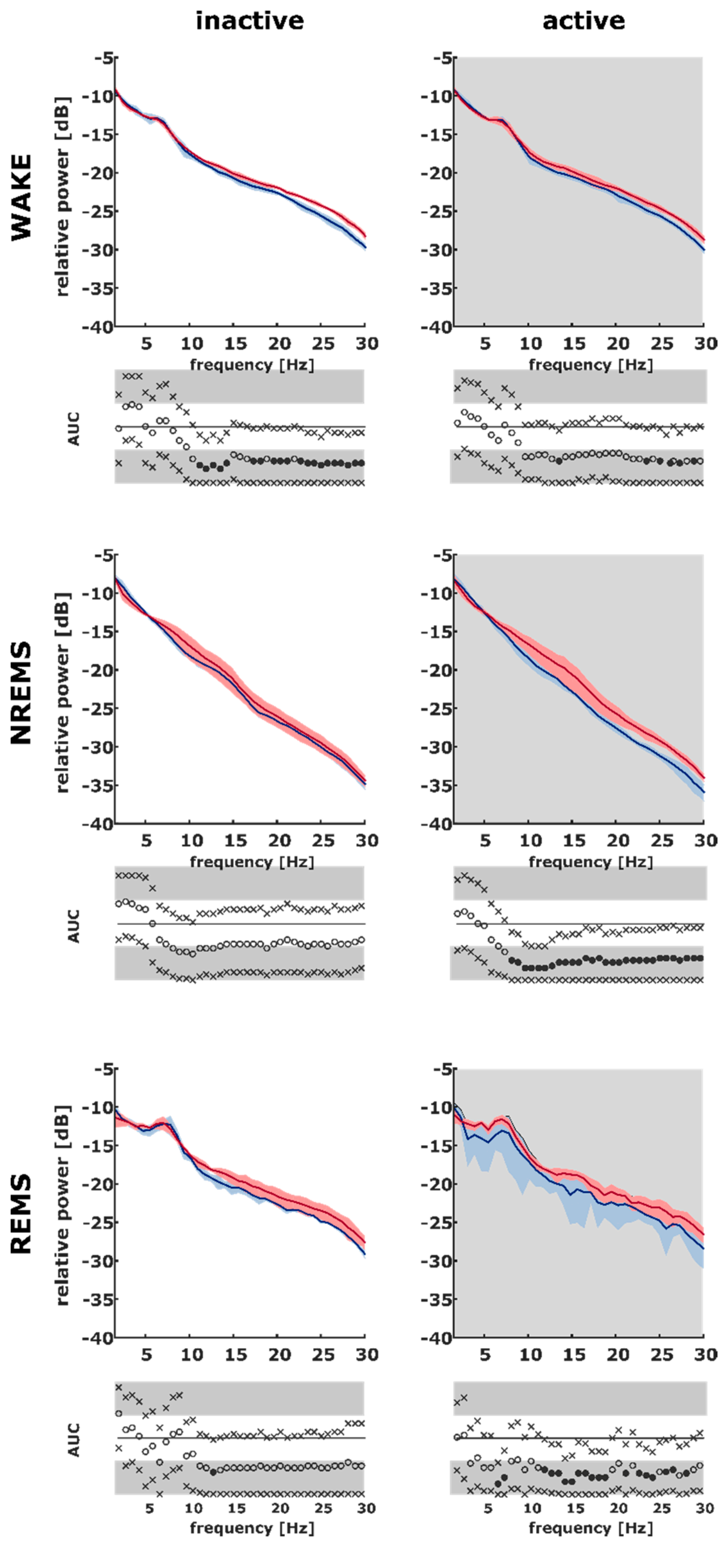

Figure S1. Plots of the median of the relative spectral power for the control group (AC, n = 7, for REM: n = 5, blue) and the Alzheimer's (AD, n = 8, for REM: n = 6, red) group as recorded from the caudal electrode at the different vigilance states, WAKE, NREM sleep (NREMS), and REM sleep (REMS), of the 1–30 Hz filtered data. The light blue and red shaded areas indicate the median absolute deviation. The accompanying AUC plots including the 95% confidence intervals represent the results of the statistical

analysis. Dots indicate the calculated AUC, x signs indicate the limits of the 95% confidence intervals. A filled dot indicates a significant difference.

Table S1. Results of the AUC analysis including 95% confidence intervals to determine differences in the EEG band power between the AD and AC rats. Bold values indicate significance and italic values indicate a possibly relevant effect with AUC>0.7 or AUC<0.3.

		Rostral		Caudal	
		Inactive	Active	Inactive	Active
**WAKE**	**delta**	**0.95 [0.79–1]**	**0.93 [0.77–1]**	0.66 [0.34–0.93]	*0.73 [0.44–0.96]*
	**theta**	**0.11 [0–0.32]**	**0.16 [0–0.43]**	0.48 [0.16–0.79]	0.50 [0.20–0.82]
	**alpha**	**0.20 [0–0.46]**	*0.25 [0–0.57]*	*0.21 [0–0.52]*	*0.25 [0.03–0.54]*
	**beta**	**0.07 [0–0.23]**	**0.09 [0–0.29]**	**0.18 [0–0.45]**	*0.25 [0–0.55]*
**NREM**	**delta**	**0.98 [0.89–1]**	**0.91 [0.68–1]**	0.70 [0.39–0.95]	**0.82 [0.57–1]**
	**theta**	**0.05 [0–0.21]**	**0.09 [0–0.32]**	0.36 [0.09–0.66]	0.30 [0.05–0.61]
	**alpha**	**0**	**0.09 [0–0.32]**	*0.27 [0.04–0.57]*	**0.13 [0–0.36]**
	**beta**	**0.11 [0–0.32]**	**0.09 [0–0.32]**	0.30 [0.05–0.61]	**0.18 [0–0.43]**
**REM**	**delta**	0.69 [0.36–1]	*0.76 [0.45–1]*	0.64 [0.29–0.95]	**0.89 [0.67–1]**
	**theta**	0.31 [0–0.64]	**0.14 [0–0.39]**	0.55 [0.19–0.88]	**0.15 [0–0.40]**
	**alpha**	0.43 [0.10–0.76]	0.49 [0.18–0.82]	0.38 [0.07–0.71]	*0.26 [0.04–0.55]*
	**beta**	*0.24 [0–0.57]*	0.41 [0–0.81]	0.33 [0–0.69]	**0.19 [0–0.46]**

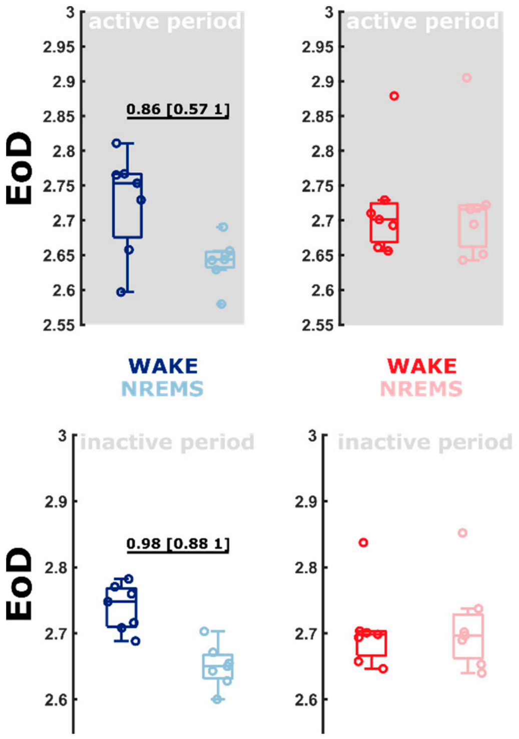

Figure S2. Comparison of entropy of difference for fast EEG dynamics between WAKE and NREM sleep. Combined box and dot plots of the entropy of difference (EoD, τ = 1) show the EEG characteristics for the comparison within groups between WAKE and NREM sleep. Blue indicates the control group (AC) and red—the Alzheimer's (AD) group. We observed significant differences (as indicated by AUC) in the AC group, but not in the AD group. The "+" indicate outlier for the box plot. NREMS: NREM sleep; REMS: REM sleep.

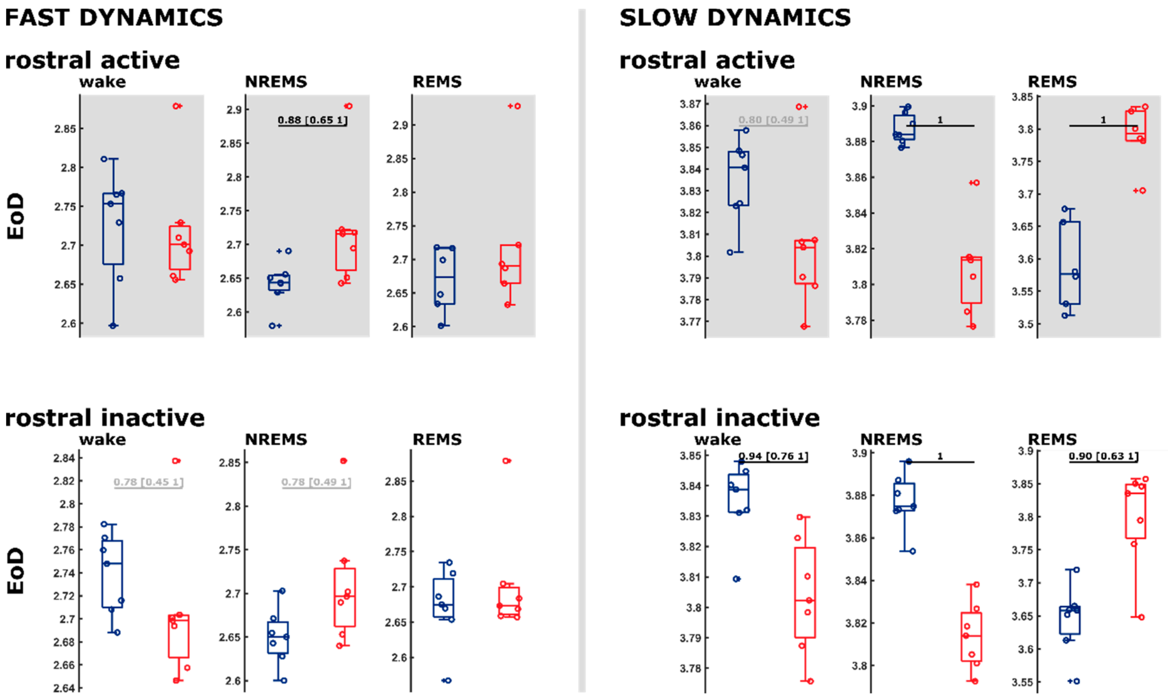

Figure S3. Entropy of difference for fast and slow EEG dynamics. Combined box and dot plots of the entropy of difference (EoD) for settings of EoD evaluating fast (τ = 1) and slow (τ = 6) EEG characteristics. Blue indicates the control group (AC) and red—the Alzheimer's (AD) group. In general, AD animals seemed to have lower (fast dynamic) EoD during WAKE (and REM sleep) as well as higher EoD during NREM sleep. Evaluation of the slow dynamic EoD revealed higher EoD in the AC group during WAKE and NREM sleep, whereas it was lower during REM sleep. Black horizontal bars indicate a significant difference between AC and AD (non-overlapping 95% confidence interval (CI) of AUC) and grey bars with grey numbers indicate overlapping CI, but AUC ≥ 0.75 indicates a relevant effect. The "+" indicate outlier for the box plot. The black numbers indicate significance, the grey numers a possibly relevant effect. NREMS: NREM sleep; REMS: REM sleep.

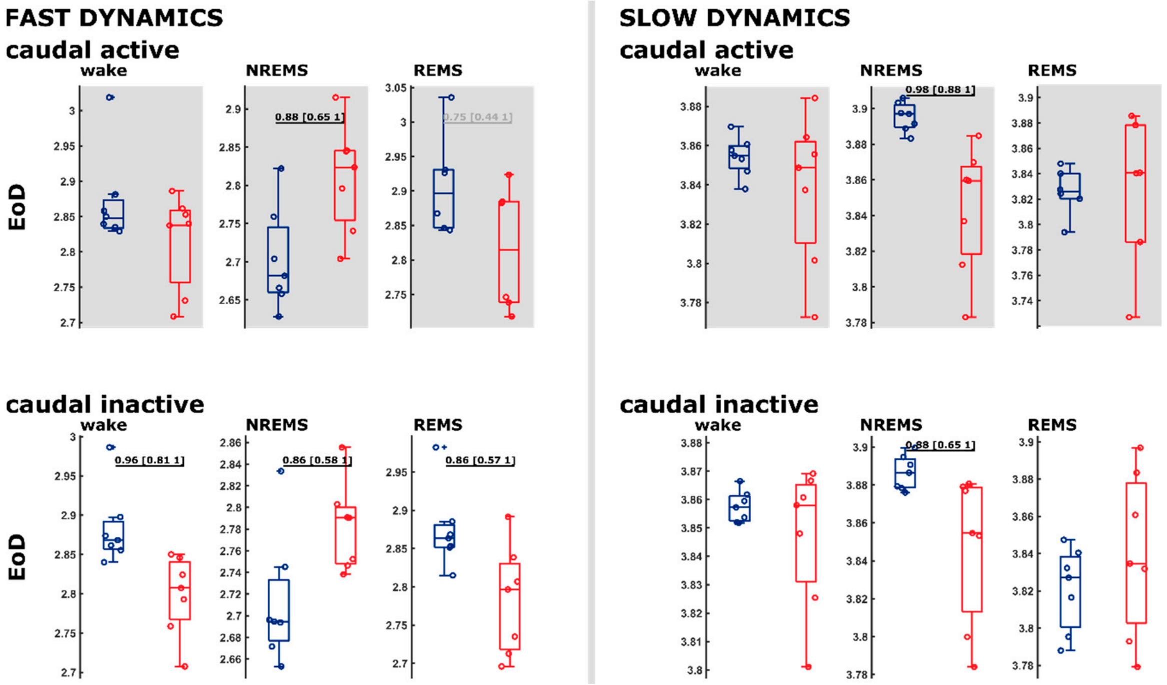

Figure S4. Entropy of difference for fast and slow EEG dynamics. Combined box and dot plots of the entropy of difference (EoD) for settings of EoD evaluating fast (τ = 1) and slow (τ = 6) EEG characteristics. Blue indicates the control group (AC) and red—the Alzheimer's (AD) group. In

general, AD animals seemed to have lower (fast dynamic) EoD during WAKE (and REM sleep) as well as higher EoD during NREM sleep. Evaluation of the slow dynamic EoD revealed higher EoD in the AC groupduring WAKE and NREM sleep, whereas it was lower during REM sleep. Black horizontal bars indicate a significant difference between AC and AD (non-overlapping 95% confidence interval (CI) of AUC) and grey bars and numbers indicate overlapping CI, but AUC ≥ 0.75 indicates a relevant effect. The "+" indicate outlier for the box plot. NREMS: NREM sleep; REMS: REM sleep.

Table S2. Results of the AUC analysis including 95% confidence intervals for the comparisons between AC and AD at different vigilance states or within the AC or AD group between WAKE and NREM sleep of the EOD with different settings, i.e., τ = 1 (fast) and τ = 6 (slow). NREMS: NREM sleep; REMS: REM sleep. Bold values indicate significance and italic values indicate a possibly relevant effect with AUC>0.7 or AUC<0.3.

	**AC vs. AD**	**WAKE**		**NREMS**		**REMS**	
		**Fast**	**Slow**	**Fast**	**Slow**	**Fast**	**Slow**
**AM**	**Rostral**	0.63 [0.31–1]	*0.80 [0.49–1]*	**0.12 [0–0.35]**	**1**	0.39 [0.08–0.75]	**0**
	**Caudal**	0.59 [0.27–0.88]	0.61 [0.29–0.91]	**0.12 [0–0.35]**	**0.98 [0.88–1]**	*0.75 [0.44–1]*	0.38 [0–0.75]
**PM**	**Rostral**	*0.78 [0.45–1]*	**0.94 [0.76–1]**	*0.22 [0–0.51]*	**1**	0.49 [0.18–0.82]	**0.1 [0–0.37]**
	**Caudal**	**0.96 [0.81–1]**	0.53 [0.18–0.86]	**0.14 [0–0.43]**	**0.88 [0.65–1]**	**0.86 [0.57–1]**	0.37 [0.08–0.69]

	**WAKE vs. NREMS**	**AC**		**AD**	
		**Fast**	**Slow**	**Fast**	**Slow**
**AM**	**Rostral**	**0.86 [0.57–1]**	**0**	0.46 [0.14–0.82]	0.39 [0.08–0.71]
	**Caudal**	**1**	**0**	0.57 [0.24–0.88]	0.43 [0.12–0.76]
**PM**	**Rostral**	**0.98 [0.88–1]**	**0**	0.51 [0.20–0.82]	0.35 [0.08–0.67]
	**Caudal**	**1**	**0**	0.65 [0.33–0.96]	0.45 [0.12–0.78]
